# Integrative analysis of DNA methylation and gene expression through machine learning identifies stomach cancer diagnostic and prognostic biomarkers

**DOI:** 10.1111/jcmm.17693

**Published:** 2023-02-13

**Authors:** Maryam Hosseini, Maryam Lotfi‐Shahreza, Parvaneh Nikpour

**Affiliations:** ^1^ Department of Genetics and Molecular Biology, Faculty of Medicine Isfahan University of Medical Sciences Isfahan Iran; ^2^ Department of Computer Engineering, Shahreza Campus University of Isfahan Isfahan Iran

**Keywords:** diagnostic biomarkers, DNA methylation, gene expression, machine learning, prognostic biomarkers, stomach cancer

## Abstract

DNA methylation is an early event in tumorigenesis. Here, by integrative analysis of DNA methylation and gene expression and utilizing machine learning approaches, we introduced potential diagnostic and prognostic methylation signatures for stomach cancer. Differentially‐methylated positions (DMPs) and differentially‐expressed genes (DEGs) were identified using The Cancer Genome Atlas (TCGA) stomach adenocarcinoma (STAD) data. A total of 256 DMPs consisting of 140 and 116 hyper‐ and hypomethylated positions were identified between 443 tumour and 27 nontumour STAD samples. Gene expression analysis revealed a total of 2821 DEGs with 1247 upregulated and 1574 downregulated genes. By analysing the impact of cis and trans regulation of methylation on gene expression, a dominant negative correlation between methylation and expression was observed, while for trans regulation, in hypermethylated and hypomethylated genes, there was mainly a negative and positive correlation with gene expression, respectively. To find diagnostic biomarkers, we used 28 hypermethylated probes locating in the promoter of 27 downregulated genes. By implementing a feature selection approach, eight probes were selected and then used to build a support vector machine diagnostic model, which had an area under the curve of 0.99 and 0.97 in the training and validation (GSE30601 with 203 tumour and 94 nontumour samples) cohorts, respectively. Using 412 TCGA‐STAD samples with both methylation and clinical data, we also identified four prognostic probes by implementing univariate and multivariate Cox regression analysis. In summary, our study introduced potential diagnostic and prognostic biomarkers for STAD, which demands further validation.

## 1 INTRODUCTION

Stomach cancer is the 5th most frequently diagnosed, the 7th most prevalent cancer and the third cause of cancer‐related deaths, worldwide.^1^, ^2^ The majority of stomach cancer patients are diagnosed at an advanced stage, resulting in a poor prognosis.^3^ Early diagnosis of this cancer is very challenging, because of its rare initial symptoms.^4^ Although some biomarkers have been proposed for detecting the early stage of stomach cancer or its progression, there is still a significant gap in identifying biomarkers with high sensitivity and specificity for effective diagnosis and prognosis of this cancer.^3^

DNA methylation is an epigenetic event that involves adding a methyl group to 5th carbon of cytosine to form 5‐methylcytosine.^5^ DNA methylation is involved in a variety of biological processes, including gene expression regulation,^6^ alternative splicing,^7^ genomic imprinting,^8^ cell differentiation,^9^ development^10^ and inflammation.^11^ Methylation abnormalities have been linked to a variety of diseases, including cancer.^12^ DNA methylation is associated with a greater number of abnormal alterations per cancer cell^13^ and it occurs earlier in the tumorigenesis of many cancer types, making it a useful tool for cancer diagnosis, risk screening, prognosis and treatment prediction.^14^

In the current study, by integrated analysis of methylation, RNA‐seq gene expression and clinical data of stomach adenocarcinoma (STAD) from The Cancer Genome Atlas (TCGA) database and applying a machine learning approach, we identified eight potential diagnostic methylation biomarkers with a precision of 0.94, recall of 0.93 and area under the curve (AUC) of 0.97 in an independent validation set from the Gene Expression Omnibus (GEO) database. We furthermore introduced four methylation probes as prognostic biomarkers for STAD and checked their performance for predicting high‐ and low‐risk patients independently from clinicopathological features.

## 2 MATERIALS AND METHODS

### 2.1 Data sources and preparation

DNA methylation and gene expression profiles for STAD were downloaded from TCGA database using TCGAbiolinks R package.^15^, ^16^ The training set was the methylation data that consisted of a dataset including 48 STAD tumour and 25 nontumour samples assayed using Illumina HumanMethylation27 BeadChip platform (27 K array) and a dataset of 395 tumour and 2 nontumour STAD samples assayed using Illumina HumanMethylation450 BeadChip platform (450 K array). Although using 450 K arrays would increase the number of methylation probes, TCGA‐STAD has only 2 nontumour samples profiled with 450 K array. By combining two different array platforms (27 K and 450 K arrays), a number of nontumour samples will be increased to 27, which is more rational in statistical analyses. So, we analysed the 25,978 CpG sites, which were common between 450 K and 27 K platforms. The methylation level of CpGs was described as beta values (*β*), which are the ratio of the intensities between methylated and total signals, ranging from 0 to 1. Annotations for CpG probes were retrieved using a R package IlluminaHumanMethylation450kanno.ilmn12.hg19 according to human reference genome assembly GRCh37 (hg19). Level 3 Illumina HiSeq_RNASeqV2 data of 450 samples (415 STAD and 35 nontumour samples) were downloaded as a source of transcriptome data. Moreover, an independent DNA methylation dataset, i.e., GSE30601 was collected from the GEO as a validation set. This dataset consisted of 203 gastric tumours and 94 matched gastric nonmalignant samples, which was profiled using an Illumina HumanMethylation27 BeadChip platform (GPL8490).

### 2.2 Differential analysis of DNA methylation

Differential methylation analysis was performed between 443 STAD tumour and 27 nontumour samples. Preprocessing was performed to remove probes containing missing values in any sample, overlapping with single‐nucleotide polymorphisms (SNPs) or locating at sex chromosomes. ChAMP package^17^, ^18^ was utilized to identify differentially‐methylated positions (DMPs). Those probes with adjusted *p*‐values < 0.05 and |delta *β*| > 0.25 were considered as DMPs.

### 2.3 Differential analysis of gene expression

We used TCGA‐STAD transcriptome data for the identification of differentially‐expressed genes (DEGs) utilizing DESeq2 R package.^19^ After normalizing the data, those genes satisfied the threshold of |log_2_ fold change (FC)| > 1.5 and adjusted *p*‐values < 0.05 were considered to be statistically significant differentially‐expressed.

### 2.4 Correlation analysis between DNA methylation and gene expression

In this study, we examined the impact of DNA methylation on both local (cis) and distant (trans) regulation of gene expression. For cis‐regulation, the Pearson correlation was calculated between *β* values of those CpGs, which resided in the promoter regions and normalized expression values of their corresponding genes. The criteria to screen out the significant correlations were |correlation coefficient| > 0.3 and adjusted *p*‐values < 0.05. Since there can be multiple CpGs in one promoter, the relationship between each CpG‐gene pair was independently evaluated in Pearson correlation calculations. In distant regulation, the Pearson correlation between *β* values of differentially‐expressed and ‐methylated genes and normalized expression of differentially‐expressed genes were explored.

### 2.5 Identification of candidate diagnostic CpG probes, model construction and evaluation

At first, those probes with Pearson correlation values greater than 0.7 with others were removed from the data. Recursive feature elimination with cross‐validation (RFECV) method^20^ was then implemented on the remaining probes to select final features for model construction. Because our training cohort was imbalanced, meaning the number of samples in tumour (T) group outnumbered the samples in nontumour (NT) group (443T vs. 27 NT), synthetic minority oversampling with Tomek link (SMOTETomek) method^21^ was applied to address the imbalance issue with generation of synthetic data. Those finally selected candidate probes were used to build a diagnostic model based on different algorithms including support vector machine (SVM), logistic regression, random forest, GaussianNB, gradient boosting, AdaBoost and decision tree. A model with the highest AUC among the others was selected for hyperparameter tuning to improve its performance. GSE30601 dataset was used for the verification of final candidate probes. To evaluate the performance of the model, a comprehensive list of metrics including AUC, F1‐score, precision, specificity, sensitivity (recall) and accuracy were used to measure the discriminative capability. Machine learning analysis and visualizations were performed using scikit‐learn^22^ and matplotlib^23^ Python libraries.

### 2.6 Prognostic biomarker selection

At first, those methylation probes with negative correlation with normalized expression values of genes were selected for subsequent analysis (correlation coefficient < −0.3 and adjusted *p*‐values < 0.05). As the negative correlation between methylation and gene expression is much well‐understood,^24^ so just those methylation probes with negative correlation with normalized expression values of genes were used in the analyses. 412 TCGA‐STAD samples with both methylation and clinical data were used for prognosis analysis. They were randomly divided into 60% training and 40% validation sets. Univariate Cox proportional hazard regression analysis was performed to select those methylation probes, which were significantly associated with the overall survival (OS) of patients with *p*‐values < 0.01. Selected CpG probes were then subjected to multivariate Cox stepwise regression analysis using a MASS R package. In the multivariate analysis, both forward and backward directions were used and the selection was based on the Akaike information criterion (AIC).^25^ A risk score formula was then constructed based on a linear combination of methylation levels of probes multiplied by their coefficients in multivariate Cox regression analysis, as follows^26^, ^27^:

$$\text{Risk score}=\sum_{i=1}^{k}\left(\beta_{i}\times S_{i}\right)$$

Where:

- k is the number of candidate probes
- $\beta_{i}$ is the coefficient index of candidate probe
- $S_{i}$ is the methylation level of candidate probe

Patients were then divided into high‐ and low‐risk groups based on the median of calculated risk score and their OS was compared by applying Kaplan–Meier analysis in an R‐based survminer package.

### 2.7 Gene set enrichment analysis

Gene set enrichment analysis (GSEA)^28^ was performed for the high‐risk and low‐risk groups in order to explore the biological pathways of CpG biomarkers. The annotated c2.cp.kegg.v7.5.1.symbols.gmt gene set was regarded as the gene set database. The criteria to screen out significant GSEA results were false discovery rate (FDR) *q*‐value < 0.05, *p*‐value < 0.01, normalized enrichment score (NES) > 1 and enrichment score (ES) > 0.6.

### 2.8 Statistical analysis

The calculations were performed in R software version 4.1.2. Machine learning sections were implemented in Python version 3.9.7 on anaconda 4.10.3. All *p*‐values were adjusted with Benjamini/Hochberg method.

## 3 RESULTS

### 3.1 Identification of STAD DMPs and DEGs

**FIGURE 1:**
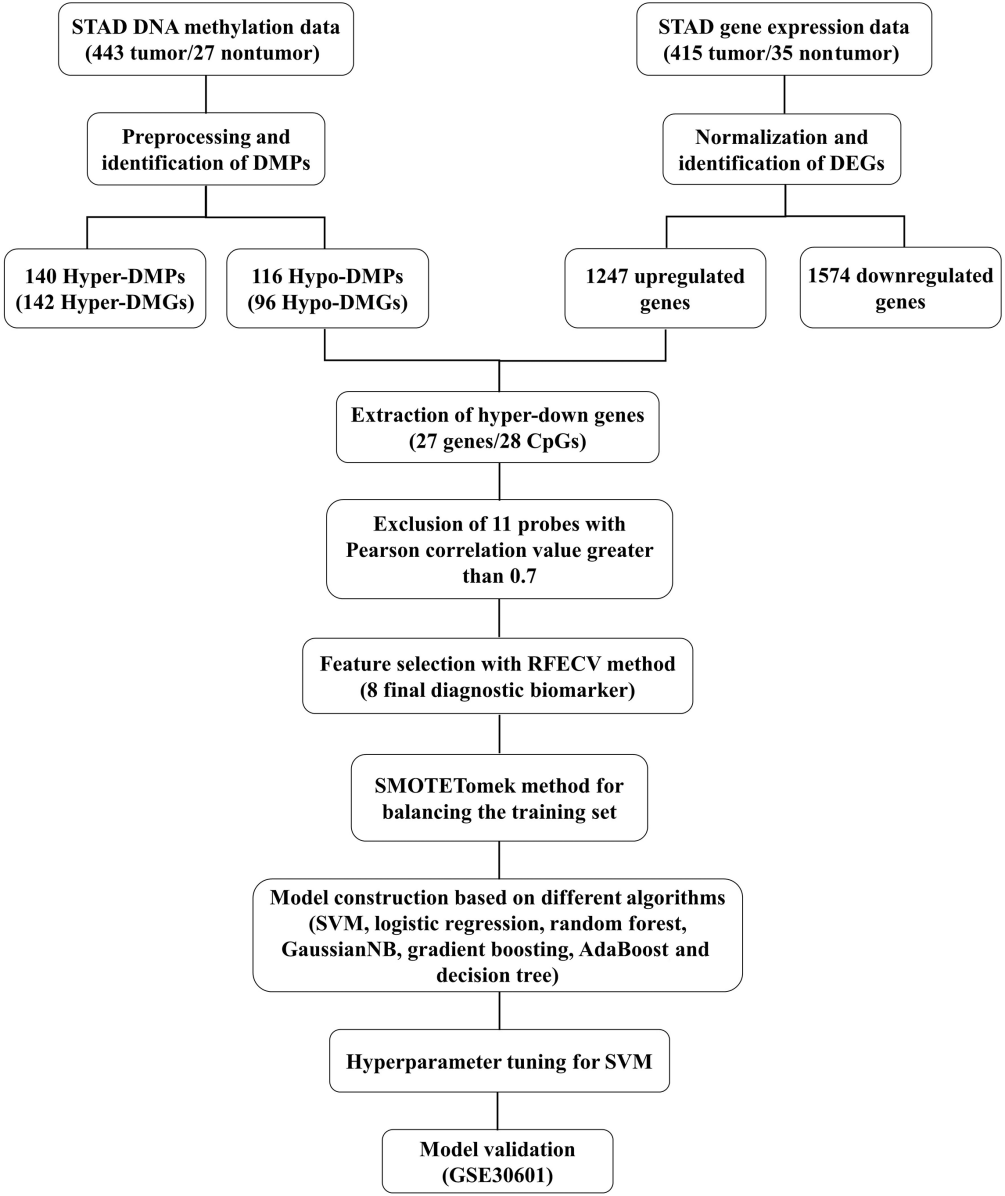
The workflow for identification of STAD diagnostic biomarkers. Hypermethylated probes locating in the promoter region of downregulated genes were used for biomarker selection. After removing correlated features, eight final methylation probe candidates were identified using RFECV. SMOTETomek method was applied to address the imbalance issue of training set, which increased the number of nontumour samples to 443. Different algorithms were compared based on their AUCs and SVM was selected as the best final model. SVM's hyperparameters were tuned and its performance was evaluated in an external validation set. AUC, area under the curve; DEGs, differentially‐expressed genes; DMGs, differentially‐methylated genes; DMPs, differentially‐methylated positions (probes); RFECV, recursive feature elimination with cross‐validation; SMOTETomek, synthetic minority oversampling with Tomek link; STAD, stomach adenocarcinoma; SVM, support vector machine.

**FIGURE 2:**
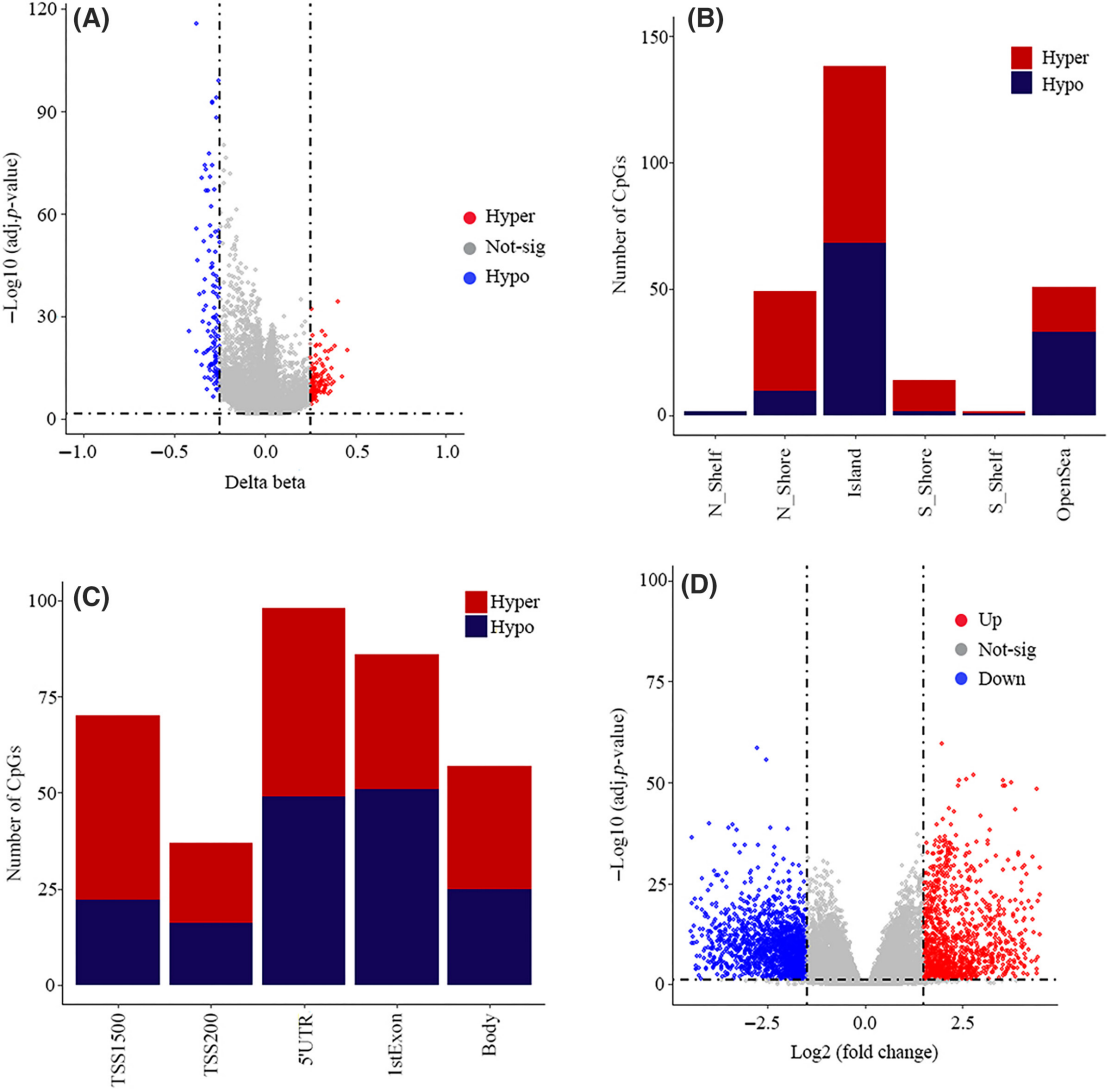
DMPs and DEGs analysis. Volcano plot for DMPs. DMPs were found with the criteria of |∆*β*| > 0.25 and adjusted *p*‐value < 0.05. Red and blue dots are hypermethyalted and hypomethylated DMPs, respectively, and the gray ones were not significant according to the above‐mentioned criteria (A). Distribution of DMPs in different genomic regions defined by the distance from CGI. Red and dark blue parts stand for hypermethylated and hypomethylated DMPs, respectively (B). Distribution of DMPs in different genomic regions defined by the distance from TSS. Red and dark blue parts represent for hypermethylated and hypomethylated DMPs, respectively (C). Volcano plot for DEGs. DEGs were found with the criteria of |log_2_ FC| > 1.5 and adjusted *p*‐value < 0.05. Red and blue dots represent upregulated and downregulated DEGs, respectively, and the grey ones were not significant according to the defined criteria (D). 1stExon, the first exon; 5′UTR, 5′ untranslated region; adj‐*p*‐value, Benjamini/Hochberg adjusted *p*‐value; CGI, CpG island; DEGs, differentially‐expressed genes; DMPs, differentially‐methylated positions (probes); Island, CpG island; N_Shelf, North Shelf (2–4 kb upstream of CGI); N_Shore, North Shore (0–2 kb upstream of CGI); Open Sea, further than 4 kb from CGI; S_Shelf, South Shelf (2–4 kb downstream of CGI); S_Shore, South Shore (0–2 kb downstream of CGI); TSS, transcription start site; TSS1500, 200–1500 nucleotides upstream of TSS); TSS200, 0–200 nucleotides upstream of TSS.

The study procedures for the identification of STAD diagnostic biomarkers are presented in Figure 1. By performing preprocessing steps, from 48,5577 probes in the primary data, 25,978 of them were identified to be common between 27 K and 450 K platforms and remained for further analysis. Then, by removing probes containing missing values in any sample, overlapping with SNPs or locating at sex chromosomes, 18,291 final probes were remained for differential methylation analysis in the next steps. We then performed differential methylation analysis on 443 tumour and 27 nontumour TCGA‐STAD samples and found a total of 256 DMPs (|∆*β*| > 0.25 and adjusted *p*‐values < 0.05), which consisted of 140 and 116 hyper‐ and hypomethylated positions, respectively (Figure 2A). Then, we sought to explore the distribution of DMPs in different genomic regions relative to CpG islands (CGIs), including North Shelf (N_Shelf, 2–4 kb upstream of CGI), North Shore (N_Shore, 0–2 kb upstream of CGI), CGI (Island), South Shore (S_Shore, 0–2 kb downstream of CGI), South Shelf (S_Shelf, 2–4 kb downstream of CGI) and Open Sea (further than 4 kb from CGI). Hypermethylated sites were mainly located in CGI, and generally, a reduction in their number could be seen with increasing the distance from CpG island (Figure 2B). We furthermore examined the distribution of DMPs in different positions relative to transcription start sites (TSSs), including TSS1500 (200–1500 nucleotides upstream of TSS), TSS200 (0–200 nucleotides upstream of TSS), 5′ untranslated region (5′UTR), first exon (1st Exon) and gene body. Hypermethylated probes were mainly observed in TSS1500 while hypomethylated sites were predominantly located in the 5′UTR and first exon (Figure 2C). In order to find differentially‐expressed genes, we next analysed gene expression data of 415 tumour and 35 nontumour TCGA‐STAD samples. Analysis of these data revealed a total of 2821 DEGs (|log_2_ FC| > 1.5 and adjusted *p*‐values < 0.05), which were divided into 1247 upregulated and 1574 downregulated genes (Figure 2D).

### 3.2 Identification of relevant DNA methylation changes associated with gene expression

**FIGURE 3:**
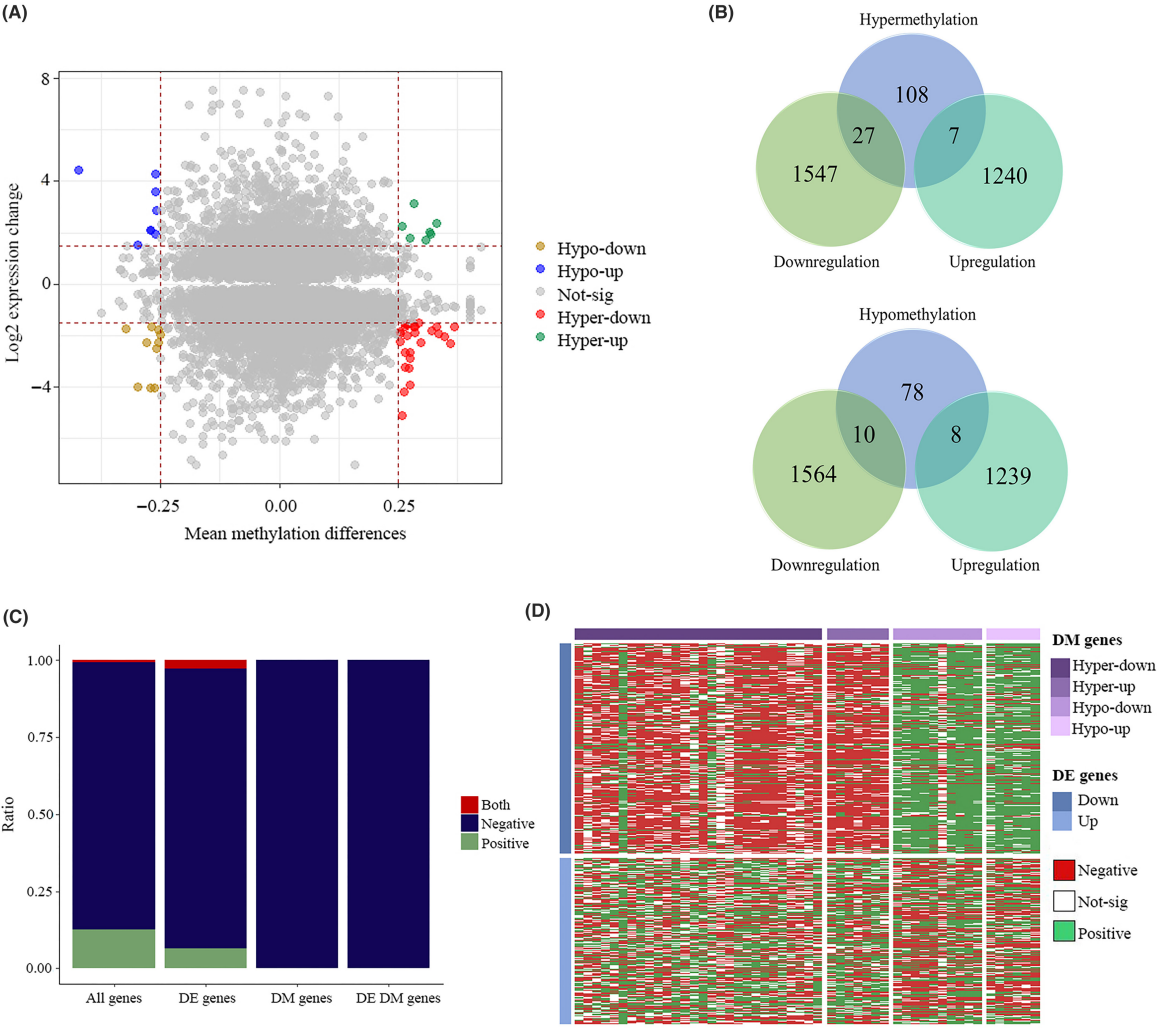
Integrative analysis of gene expression and DNA methylation. Scatterplot of mean methylation differences versus log_2_ expression fold change. Each point belongs to a specific CpG‐gene pair. Based on the mean methylation differences and log_2_FCs, genes were classified into four groups including: hypomethylated‐downregulated (yellow), hypomethylated‐upregulated (blue), not significant (gray), hypermethylated‐downregulated (red) and hypermethylated‐upregulated (green) (A). Venn diagram showing the intersection between hypermethylated genes and DEGs (top) and hypomethylated genes and DEGs (bottom) (B). Local regulation of a gene's expression by its own promoter methylation. Pearson correlation was investigated for all genes, DEGs, DMGs and differentially‐expressed and ‐methylated genes. Those relationships with |correlation coefficient| > 0.3 and adjusted *p*‐value < 0.05 were considered as significant. In all cases a dominant negative correlation was observed (C) Trans regulation of gene expression by promoter methylation. Pearson correlation was calculated between 51 differentially‐expressed and ‐methylated genes and 2821 DEGs. Hypermethylated and hypomethylated genes showed a predominantly negative and positive correlation with gene expression of DEGs (D). DE DM genes, differentially‐expressed and ‐methylated genes; DE genes, differentially‐expressed genes; DEGs, differentially‐expressed genes; DM genes, differentially‐methylated genes; DMGs, differentially‐methylated genes.

We next investigated the intersection between differentially‐methylated genes (DMGs) and DEGs. Genes were considered to be DMGs, if there was at least one differentially‐methylated probe in their promoter region, including TSS1500, TSS200, 5′UTR and first exon. The common genes were classified into four groups named: hypermethylated‐upregulated (hyper‐up), hypermethylated‐downregulated (hyper‐down), hypomethylated‐upregulated (hypo‐up) and hypomethylated‐downregulated (hypo‐down) (Figure 3A and Table S1). Among 142 hypermethylated genes, 27 and 7 of them showed a downregulation and upregulation pattern, respectively, and from 96 hypomethylated genes, 10 and 8 of them were down‐ and upregulated, respectively (Figure 3B). Because aberrant hypermethylation of promoter is a critical epigenetic phenomenon in cancer and it impacts important genes and mechanisms related to cancer pathogenesis, like inactivation of tumour suppressor genes,^29^, ^30^ in this study we just included those genes with simultaneous hypermethylated and downregulated pattern for subsequent analysis and biomarker identification.

Correlation analysis is a common approach to study the relationship between DNA methylation and gene expression.^31^, ^32^ Here, we investigated the impact of DNA methylation on both local (cis) and distant (trans) regulation of gene expression. In cis‐regulation, the main goal was to explore the impact of promoter methylation of one gene on its own expression. In total, 18,459 CpG‐gene pairs were analysed in which 207 among them showed a significant positive correlation, 1413 showed a significant negative correlation, and as multiple CpGs can be found in the promoter of one gene, 13 of genes showed both significant positive and negative correlations. A dominant negative correlation was also observed in correlation analysis between DEGs, DMGs and genes with simultaneous differential expression and methylation and CpGs (Figure 3C).

In order to assess the association between the expression of a gene and the promoter methylation status of other genes, a Pearson correlation analysis was conducted between 51 CpG probes associated with genes showing simultaneous differential expression and methylation and 2821 DEGs. Results revealed that hypermethylated genes had predominantly a negative correlation with gene expression while hypomethylated genes were more likely to be positively correlated with the expression of DEGs (Figure 3D).

### 3.3 Identification of diagnostic biomarkers for STAD

**FIGURE 4:**
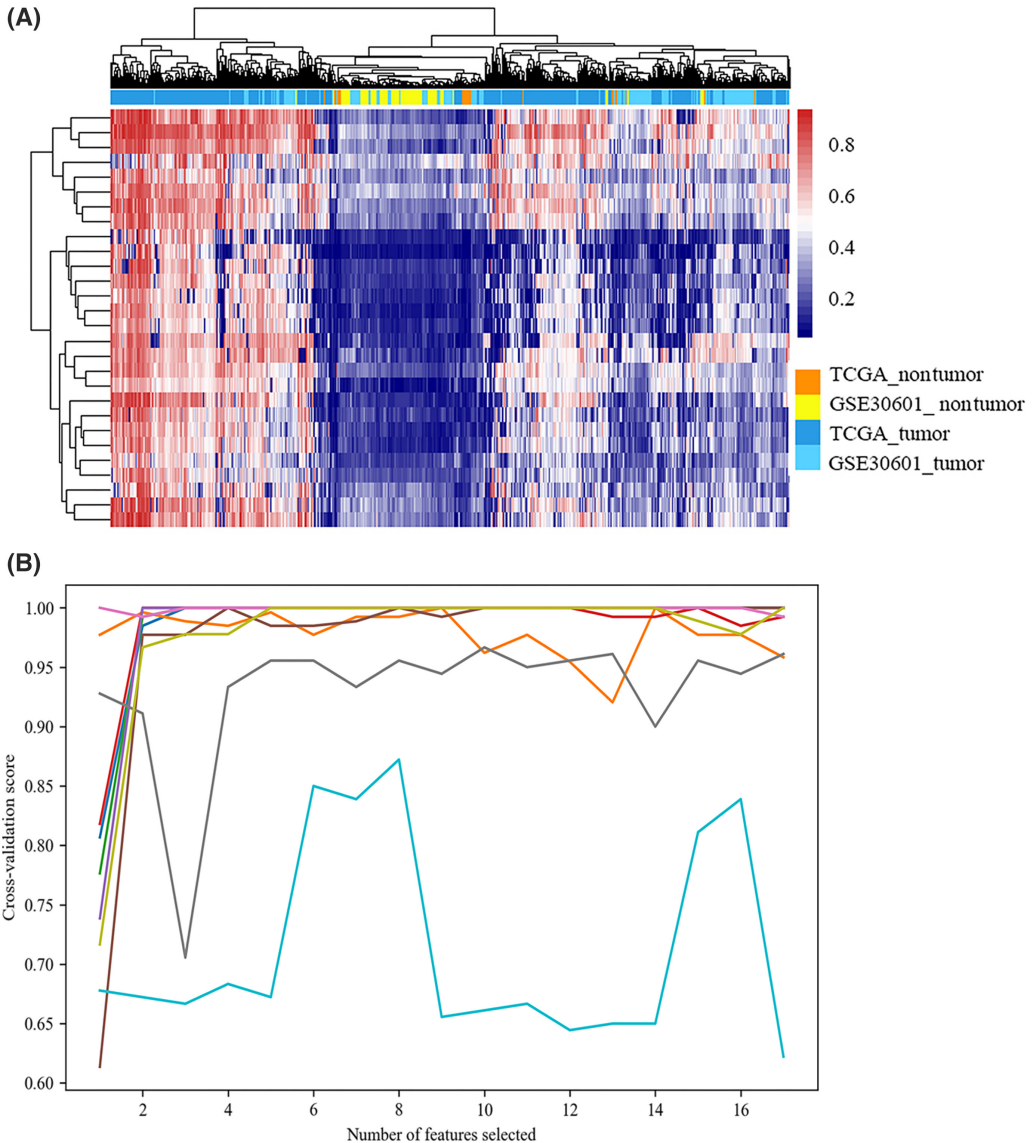
Identification of STAD diagnostic biomarkers. Hierarchical clustering of 28 hypermethylated and downregulated probes in 443 tumour and 27 nontumour samples in TCGA‐STAD methylation data and 203 tumour and 94 nontumour samples from GSE30601. Tumour and nontumour samples were clustered together based on these candidate probes (A). Feature selection with RFECV and 10‐fold cross‐validation. Each colour represents one‐fold of cross‐validation with checking different number of features on *X* axis and validation score on *Y* axis (B). RFECV, recursive feature elimination with cross‐validation; STAD, stomach adenocarcinoma; TCGA, The Cancer Genome Atlas.

In order to find diagnostic biomarkers for STAD, we started with 28 hypermethylated probes locating in the promoter region of 27 downregulated genes. Hierarchical clustering of these 28 probes from both TCGA and GSE30601 methylation data, showed that tumour and nontumour samples could be well‐separated from each other based on these probes (Figure 4A). Of these 28 candidate probes, we selected those which had the Pearson correlation coefficient of less than 0.7. This correlation‐based filtration resulted in removing 11 probes out of those first 28 candidate probes. The recursive feature elimination with the 10‐fold cross‐validation (RFECV) method was then used to further filter out and evaluate the discriminative power of those 17 remaining candidate probes. RFECV selected 8 probes as final candidate probes (Figure 4B). Table 1 shows the features of these 8 final probes.

**TABLE 1:** Characteristics of 8 selected candidate probes for the diagnostic model

Probe	Chromosome	Gene	Location
cg17105014	chr 2	*GYPC*	TSS1500/N_Shore
cg23273897	chr 3	*MME*	TSS1500/N_Shore
cg22083047	chr 3	*PRICKLE2*	TSS1500/OpenSea
cg01049530	chr 4	*BMP3*	TSS200/Island
cg18237405	chr 6	*CPNE5*	TSS1500/Island
cg12741420	chr 6	*IRF4*	5′UTR/Island
cg11754206	chr 8	*KCNB2*	TSS1500/N_Shore
cg09396217	chr 8	*ANGPT1*	TSS200/OpenSea

Abbreviations: 5′UTR, 5′ untranslated region; Island, CpG island; N_Shore, North Shore (0–2 kb upstream of CpG island); Open Sea, further than 4 kb from CpG island; TSS, transcription start site; TSS1500, 200–1500 nucleotides upstream of TSS; TSS200, 0–200 nucleotides upstream of TSS.

### 3.4 Model construction and evaluation

**FIGURE 5:**
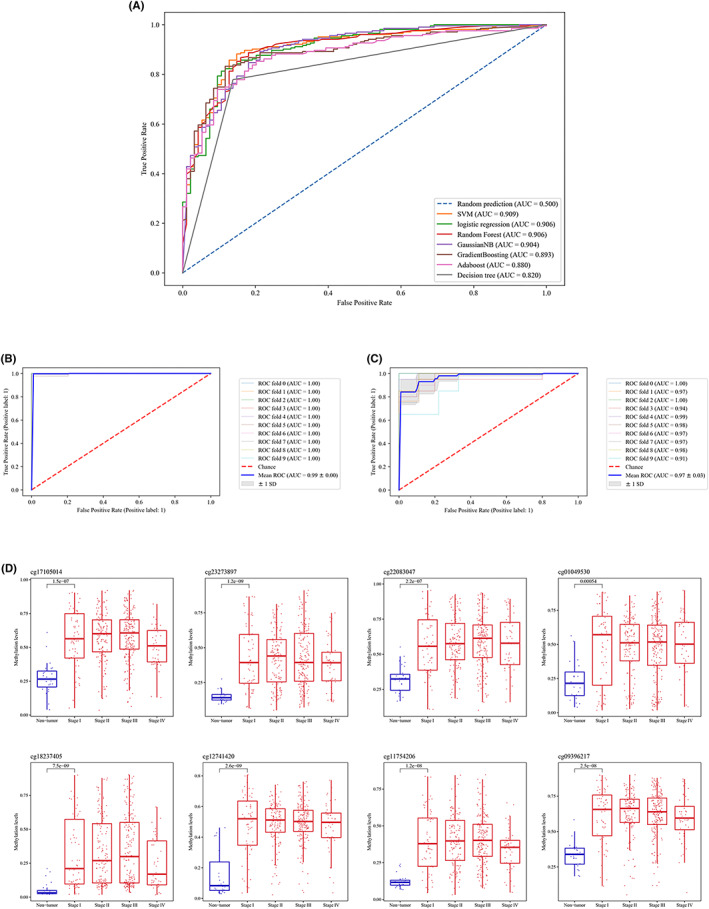
Model construction and evaluation. Comparing the performance of 7 different algorithms based on their AUCs and methylation data of 8 final candidate probes. Since SVM had the best AUC, it was selected for next steps (A). Evaluation the SVM model's performance on training set with 10‐fold cross‐validation. AUC of the model was 0.99 (B). Evaluation of the SVM model's performance on the validation cohort with 10‐fold cross‐validation. AUC of the model was 0.97 (C). Comparing the methylation level of 8 final candidate probes between nontumour (blue boxes) and different stages of tumour samples (red boxes) (D). AUC, area under the curve; SVM, support vector machine.

For model construction, TCGA methylation data were used as a training set. After implementing SMOTETomek oversampling method, the number of nontumour samples was increased to 443. Several algorithms were used to build a diagnostic model, using the new balanced training set (443T vs. 443 NT) and the 8 final candidate probes. The performance of these algorithms were compared based on their AUCs (Figure 5A). Since the SVM model had the best AUC among all others, it was selected for further analysis. SVM hyperparameters were tuned to improve their performance. Radial basis function kernel (RBF), gamma and C equal to 8.5 and 8.365 were the selected SVM hyperparameters for the final model, based on RandomizedSearchCV method in python.^33^ Model performance was evaluated on the training set with 10‐fold cross‐validation. In 10‐fold cross‐validation, data is randomly divided into 10 partitions. Each time, nine partitions will be used for training the model and one for checking its performance. Based on this approach, the AUC of the model was 0.99, which shows its great ability to distinguish STAD tumour and nontumour samples (Figure 5B). To investigate the reproducibility of the results, we furthermore checked the model's performance on an external validation set (GSE30601) with 10‐fold cross‐validation (Figure 5C). Results of this analysis were consistent with the training set and the AUC of the model on validation set was 0.97 showing that these 8 final candidate probes and the constructed SVM model have the ability to split STAD samples into tumour and nontumour ones. Besides AUC, other performance metrics including F1‐score, precision, specificity, sensitivity (recall) and accuracy are also provided in Table 2. To make sure that these potential biomarkers are suitable for the early detection of STAD, we compared their methylation levels between different tumour stages and nontumour samples. All of them were significantly hypermethylated in stage I tumours compared with nontumour samples (Figure 5D).

**TABLE 2:** Evaluation of diagnostic model's performance on the training and validation sets

Metric	Training set with 10‐fold cross‐validation	GSE30601 with 10‐fold cross‐validation
AUC	0.99	0.97
F1 score	0.99	0.93
Precision	1	0.94
Specificity	1	0.86
Sensitivity (Recall)	0.98	0.93
Accuracy	0.99	0.91

Abbreviation: AUC, area under the curve.

### 3.5 Identification of prognostic biomarkers in STAD

**FIGURE 6:**
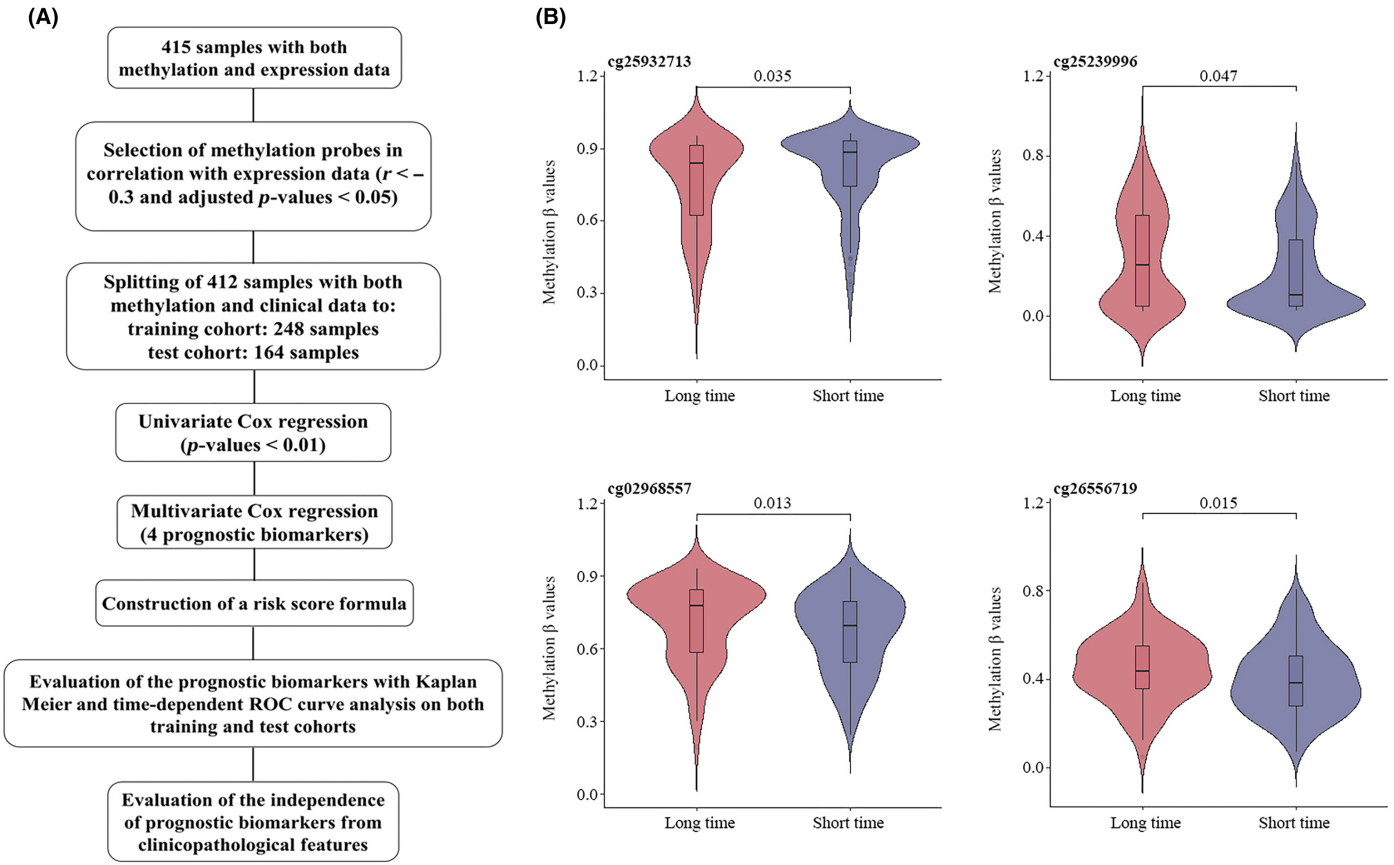
Identification of STAD prognostic biomarkers. The workflow for finding STAD prognostic biomarkers. Eight potential prognostic biomarkers were obtained using univariate Cox hazard regression and multivariate Cox stepwise regression, on the training cohort (248 TCGA‐STAD samples). The risk score formula was constructed and model's performance was evaluated using the test cohort (168 TCGA‐STAD samples) (A). Violin plots of methylation *β* values in short time (OS < 1.669) and long time (OS > 1.669) survival patients in the training cohort. The Wilcoxon test was used to determine the difference between two groups. *p*‐Values are shown at the top of each plot (B). OS, overall survival; *r*, correlation coefficient; ROC, receiver‐operating characteristic; STAD, stomach adenocarcinoma; TCGA, The Cancer Genome Atlas.

Figure 6A shows the workflow of the current study to find STAD prognostic biomarkers. A total of 1605 methylation probes were screened out for the next steps, based on the criteria previously mentioned. TCGA‐STAD methylation data were partitioned into training and validation cohorts at a ratio of 0.6:0.4 (248 training and 164 validation samples). Univariate Cox hazard regression analysis was implemented on training data, which showed 40 probes were significantly (*p*‐value < 0.01) associated with OS of TCGA‐STAD patients. These 40 probes were used as inputs for multivariate Cox stepwise regression, and finally, four probes were selected to be in the final model (Table 3). The risk scoring formula for the prognostic model is presented as Equation 1:

(1) $$\begin{array}{cc} \text{Risk score}= & \left(1.853917\times \mathrm{cg}25932713\right)-\left(1.027996\times \mathrm{cg}25239996\right)\\ & -\left(1.468624\times \mathrm{cg}26556719\right)-\left(1.570454\times \mathrm{cg}02968557\right). \end{array}$$

According to the risk score formula, hypermethylation of all these probes except cg25932713 was associated with longer OS in TCGA‐STAD patients. At the same time, we divided the training cohort samples into two groups based on the mean survival times (1.669). If the survival time of patients was lower than 1.669, they were classified as short time, and if it was greater than 1.669, they were referred to as long time. All the four probes showed a significant difference in their methylation status between short and long time patients (*p*‐value < 0.05) (Figure 6B). cg25932713 had a positive coefficient in multivariate Cox regression analysis and consistent with that result, here it showed a lower methylation level in long time patients, as well. On the other hand, three probes with negative coefficients in multivariate Cox regression analysis (cg25239996, cg26556719 and cg02968557) exhibited lower methylation levels in short time patients.

**TABLE 3:** Characteristics of prognostic probes in the STAD combined model

Probe	HR	CI (lower)	CI (upper)	*Z*	*p*‐Value
cg25932713	6.3847823	1.549	26.32	2.565696	0.010296889
cg25239996	0.3577230	0.135	0.95	−2.064939	0.038928819
cg26556719	0.2302421	0.062	0.85	−2.205655	0.027408159
cg02968557	0.2079507	0.066	0.66	−2.681515	0.007328952

Abbreviations: CI, confidence interval; HR, hazard ratio; STAD, stomach adenocarcinoma.

### 3.6 Assessing the prognostic potential of four candidate probes in the training and validation sets

**FIGURE 7:**
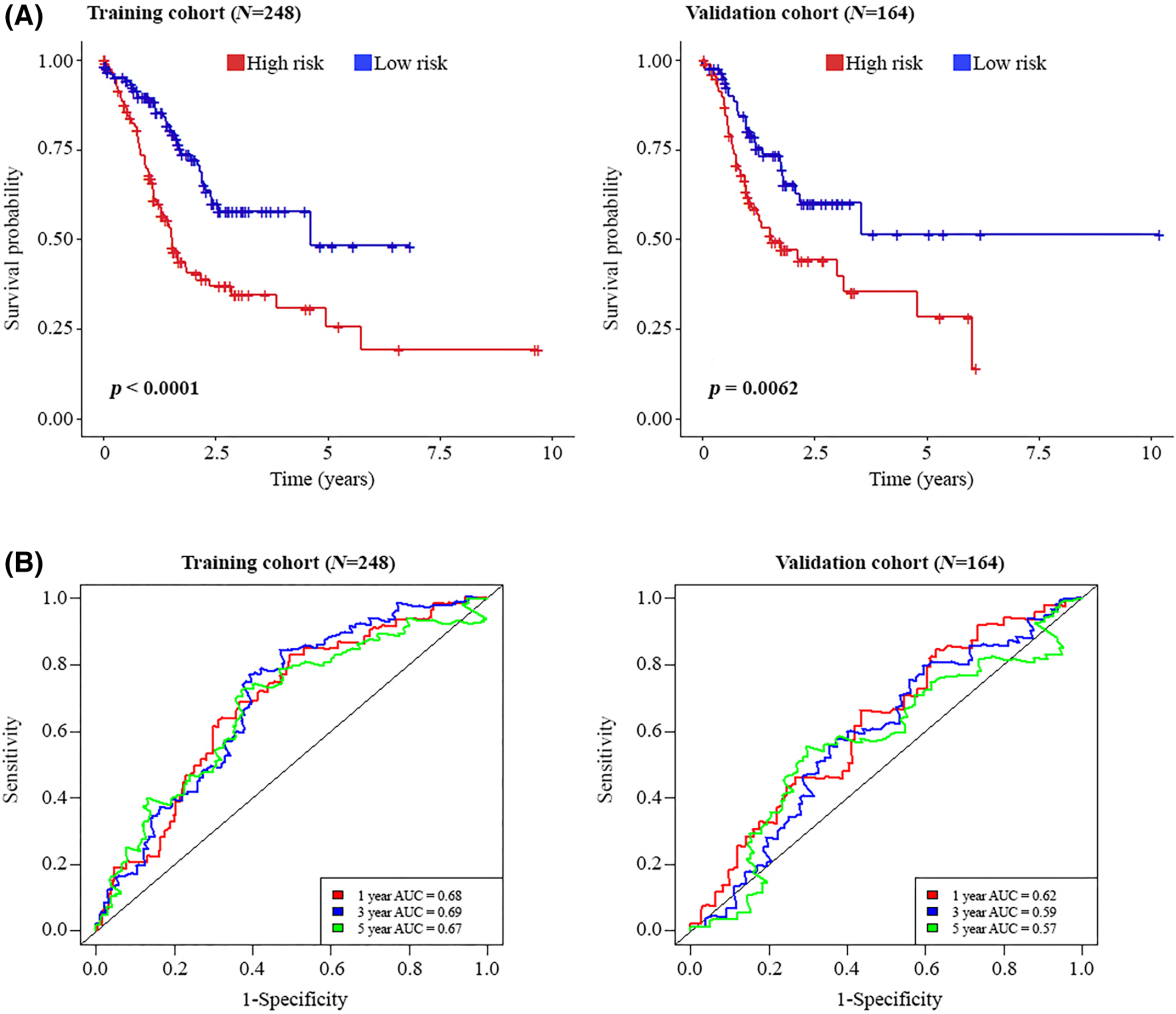
Assessing the prognostic potential of four candidate prognostic biomarkers. Kaplan–Meier analysis for low‐risk and high‐risk patients on the training and validation cohorts. Samples were divided into two groups based on the median of the risk score. In both training and validation cohorts, the low‐risk group had longer OS than the high‐risk group (A). ROC analysis for evaluation the sensitivity and specificity of 4 prognostic biomarkers (B). AUC, area under the curve; OS, overall survival; ROC, receiver‐operating characteristic.

To assess the discriminative power and accuracy of these four prognostic biomarkers, first Kaplan–Meier plots were generated for training and validation sets and patients were classified as low‐ and high‐risk groups according to the median of the calculated risk score (Figure 7A). In both training and validation cohorts, low‐risk patients had longer OS than the high‐risk group and this difference was statistically significant (*p*‐values < 0.01). We furthermore evaluated the performance of the combined model via the generation of time‐dependent ROC curves (Figure 7B). The AUC values at different times (1, 3 and 5 years follow‐ups), for training and validation sets were greater than 0.67 and 0.57, respectively.

### 3.7 Independence of the STAD prognostic model in OS prediction

In order to evaluate the independence of four introduced prognostic biomarkers from clinicopathological features like age, gender and American Joint Committee on Cancer (AJCC) stage (according to versions 6 and 7, provided in TCGA clinical data), we stratified patients based on these characteristics. Moreover, for assessing the independence from the stage, patients were grouped as an early stage if they had stage I or II and late stage if they had stage III or IV. For all these clinical features, Kaplan–Meier analysis showed a significant extension in overall survival between high‐ and low‐risk groups, and AUC values of time‐dependent ROC curve analysis also showed a distinction between these two groups as well (Figure S1).

### 3.8 GSEA analysis

In order to evaluate the mechanisms by which these four introduced prognostic biomarkers working throw, we conducted GESA analysis on the samples, which were divided into low‐ and high‐risk groups based on the median of the risk score. We found that pathways like the extracellular matrix (ECM) receptor interaction, glycosaminoglycan biosynthesis chondroitin sulfate and glycosphingolipid biosynthesis ganglio‐series were significantly correlated with gene expression data of the high‐risk group (Figure S2).

## 4 DISCUSSION

In the present study, we systematically analysed DNA methylation and gene expression data of STAD from TCGA cohort. We first identified differentially‐methylated and ‐expressed genes and then examined the correlation between DNA methylation and gene expression. We observed that there was a general negative correlation between methylation and gene expression in the context of cis‐regulation. Abnormal increased DNA methylation in normally unmethylated gene promoter CpG islands and associated gene silencing are the most well‐characterized epigenetic alterations in cancer.^29^, ^34^ Our findings were consistent with these general characteristics of tumour DNA methylation pattern. Predominant negative correlation of the methylation of hypermethylated genes with gene expression while the most likely positive correlation of the methylation of hypomethylated genes with gene expression, has been recently reported in cervical cancer^31^ and hepatocellular carcinoma^32^ in the distant (trans) regulation of gene expression. In agreement with these studies,^31^, ^32^ we also observed that in the trans regulation, hypermethylated genes mainly had a negative correlation with gene expression, but on the contrary, hypomethylated genes were more likely to be positively correlated with gene expression. The exact mechanism behind the distant impact of promoter methylation on gene expression is not yet fully elucidated.^31^

To find diagnostic biomarkers for STAD, we selected hypermethylated probes locating in the promoter region of downregulated genes for further analysis. We then removed highly correlated features, as having correlated features can make it difficult for an algorithm to generalize and this can result in biomarker redundancy.^35^ RFECV method resulted in the selection of eight probes with potential diagnostic abilities. For model construction, TCGA‐STAD methylation data (including 443 tumour and 27 nontumour samples) was used as the training set. Imbalanced data sets cause several challenges in the process of machine learning including making the model to be biased towards the majority class or even completely ignoring the minor class.^36^ We therefore implemented a method, called SMOTETomek to overcome this issue. In the next step, several machine learning algorithms were utilized to build a diagnostic model, using the new balanced training set and the eight candidate probes. SVM showed the best AUC compared with other algorithms. SVM is a powerful algorithm for model construction, which creates a hyperplane between groups in a way that it is as far as possible from borderline observations of each class.^37^ Performance of the SVM model on an external validation set showed an AUC of 0.97, which were consistent with that obtained from the training set.

The eight probes extracted from the RFECV method showing discriminative power between tumour and nontumour samples included cg17105014 (*GYPC*), cg23273897 (*MME*), cg22083047 (*PRICKLE2*), cg09396217 (*ANGPT1*), cg01049530 (*BMP3*), cg18237405 (*CPNE5*), cg12741420 (*IRF4*) and cg11754206 (*KCNB2*). Some of these genes were previously reported to be involved in the pathogenesis of gastric cancer (GC). In a study conducted by Yu et al.,^38^ hypermethylation of *GYPC* was shown to be negatively correlated with the expression of *GNAI2*, PI3K/AKT signalling pathway activation and GC cell proliferation and migration. Stromal expression of MME (also known as CD10) in primary GC is tightly correlated with invasion and metastasis and it may play a crucial role in GC pathogenesis.^39^ There is a positive correlation between PRICKLE2 expression and activation of NOTCH/WNT pathway in GC, and PRICKLE2 overexpression has been shown to be accompanied by increased invasion and poor overall survival in GC patients.^40^ Through the Akt/Survivin signalling pathway, ANGPT‐1 promotes vascular maturation and stabilizes the newly‐formed vessels.^41^ *ANGPT‐1* has been introduced as one of the independent prognostic predictors in GC.^42^ Hypermethylation of *BMP3* promoter has been reported in GC.^43^ Analysis of the RNA sequencing data of the TCGA‐STAD cohort showed that the expression of *BMP3* in gastric cancer tissues is significantly decreased compared with normal tissue and its higher expression in the primary GC tumours is associated with poorer overall survival. BMP3 is negatively correlated with cell proliferation and cell cycle‐promoting factors suggesting its inhibitory role in the proliferation of GC cells.^44^ For the three remaining probes (genes), there are not much evidence about their role in GC. However, their involvement in cancer pathogenesis has been reported in other neoplasms.^45^, ^46^, ^47^

Four probes (cg25932713, cg25239996, cg26556719 and cg02968557) were introduced as potential STAD prognostic biomarkers by implementing univariate and multivariate Cox regression analysis. cg25932713 is mapped to two DNA repair‐associated genes named *PARP9* and *DTX3L*, which were among the 10 most significantly downregulated genes in CDH1 mutated diffuse GC.^48^ Our results are in agreement with the findings of a study showing that hypermethylation of *CD40* gene, which cg25239996 maps to it, is significantly associated with the high survival rate of GC patients.^49^ Currently, there is not much evidence to support the involvement of cg26556719 and cg02968557 in GC.

Gene set enrichment analysis for these four introduced prognostic biomarkers showed a significant correlation between gene expression data of the high‐risk group and pathways like ECM receptor interaction, glycosaminoglycan biosynthesis chondroitin sulfate and glycosphingolipid biosynthesis ganglio series. ECM as the noncellular part of tissues is one of the major components of tumours that plays multiple crucial roles during tumorigenesis, including mechanical support, modulation of the microenvironment and a source of signalling molecules.^50^ For example, ECM is a suitable niche for cancer stem cell proliferation and adhesion.^51^ Glycosaminoglycans (GAGs) can be involved in multiple cancer‐related events like angiogenesis, metastasis and invasion via interaction with different factors like cytokines and growth factors.^52^ Glycosphingolipids are a subtype of glycolipids, which are the major components of surface membranes in all animal cells. They are classified into three groups named ganglio‐series, lacto‐series and globo‐series based on the type of their oligosaccharide chains. Their abnormal expression has been observed in different cancer types including gastric cancer.^53^

It should be noted that the current study has a number of limitations. First, the majority of the population in the TCGA database is Latino and Caucasian, hence evidence supporting the generalization of the present study's findings to other ethnic groups is required. Second, the prognostic analyses of this study were based on a retrospective cohort, so a prospective multicenter validation is needed before using the introduced prognostic probes as biomarkers. Third, additional in vivo and in vitro experiments are required to clarify the molecular mechanisms behind the aetiology of STAD with respect to those eight diagnostic and four prognostic methylation probes, introduced in the current study.

In summary, DNA methylation plays crucial roles in cancer development. In the current study, utilizing machine learning approaches on TCGA‐STAD data, we identified a set of eight methylation probes, being differentially‐methylated in tumour compared with nontumour tissues, which can be used in future for the early detection of STAD. We furthermore found four prognostic probes including cg25932713, cg25239996, cg26556719 and cg02968557. ROC and Kaplan–Meier analysis for these diagnostic and prognostic biomarkers showed their high sensitivity and specificity. Further experimental evaluations and clinical validations are needed for the approval of these candidate biomarkers.

## AUTHOR CONTRIBUTIONS

**Maryam Hosseini:** Data curation (lead); formal analysis (equal); investigation (equal); methodology (equal); software (lead); visualization (lead); writing – original draft (equal); writing – review and editing (equal). **Maryam Lotfi‐Shahreza:** Formal analysis (equal); methodology (equal); validation (equal); writing – review and editing (equal). **Parvaneh Nikpour:** Conceptualization (lead); funding acquisition (lead); investigation (equal); project administration (lead); resources (lead); supervision (lead); writing – original draft (equal); writing – review and editing (lead).

## CONFLICT OF INTEREST STATEMENT

The authors confirm that there are no conflicts of interest.

## Supporting information


Figure S1.

Figure S2.

Table S1.

Appendix S1.

## Data Availability

The codes used to perform the analysis are available in the Mendeley Data repository, at https://doi.org/10.17632/dcdy79pjcw.1. The results published or shown here are in whole or part based on data generated by the TCGA Research Network (https://portal.gdc.cancer.gov/legacy‐archive/search/f) and GEO (https://www.ncbi.nlm.nih.gov/geo/). GEO data can be accessed with the unique identifier GSE30601.
