# Supplementary figures and images for: Integrative analysis of DNA methylation and gene expression through machine learning identifies stomach cancer diagnostic and prognostic biomarkers
Source: J Cell Mol Med. 2023 Feb 13;27(5):714–26. doi: 10.1111/jcmm.17693 (PMC9983314; doi:10.1111/jcmm.17693)

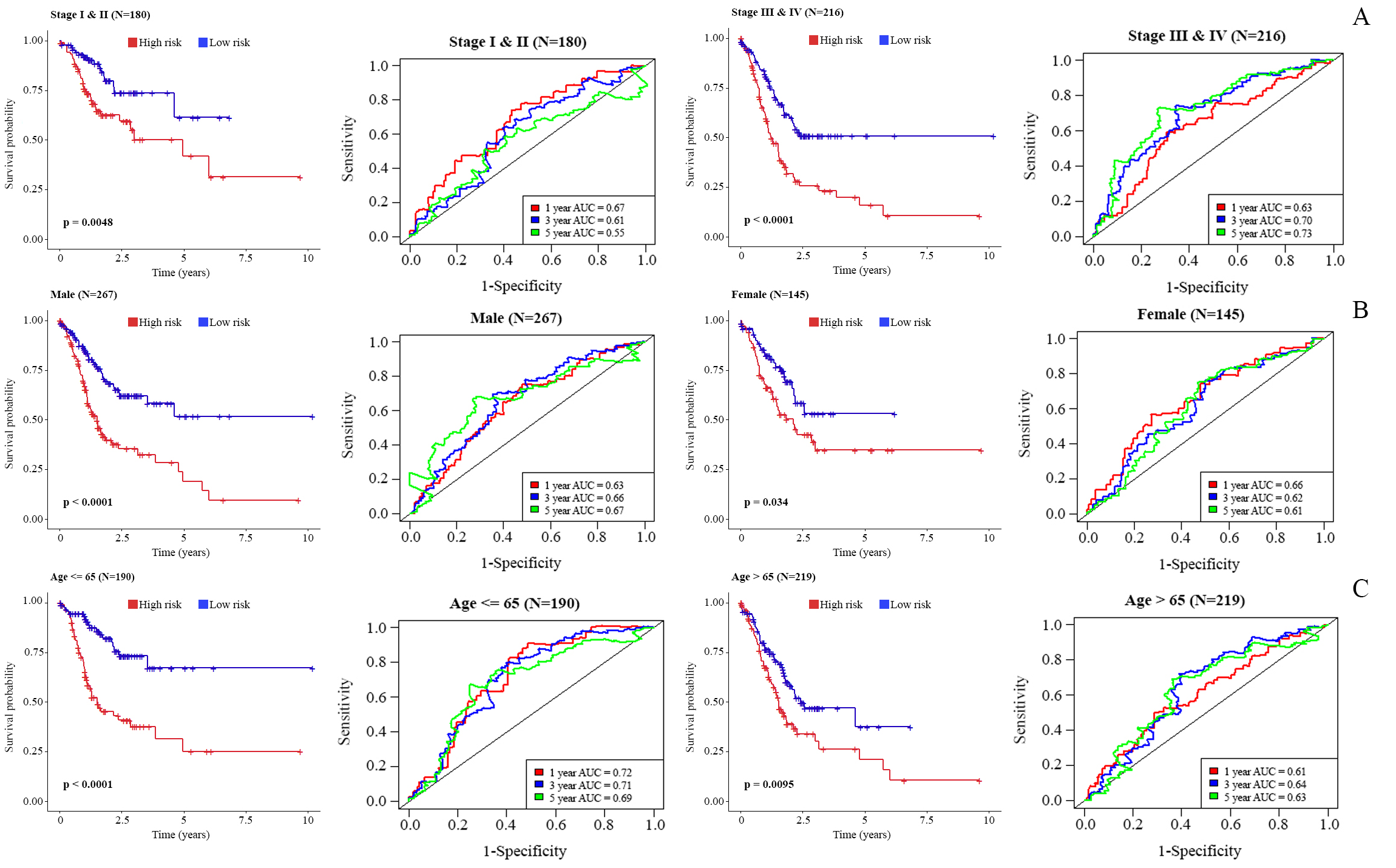

Supplement: Supplementary file 1 — Figure S1. [file JCMM-27-714-s003.png]

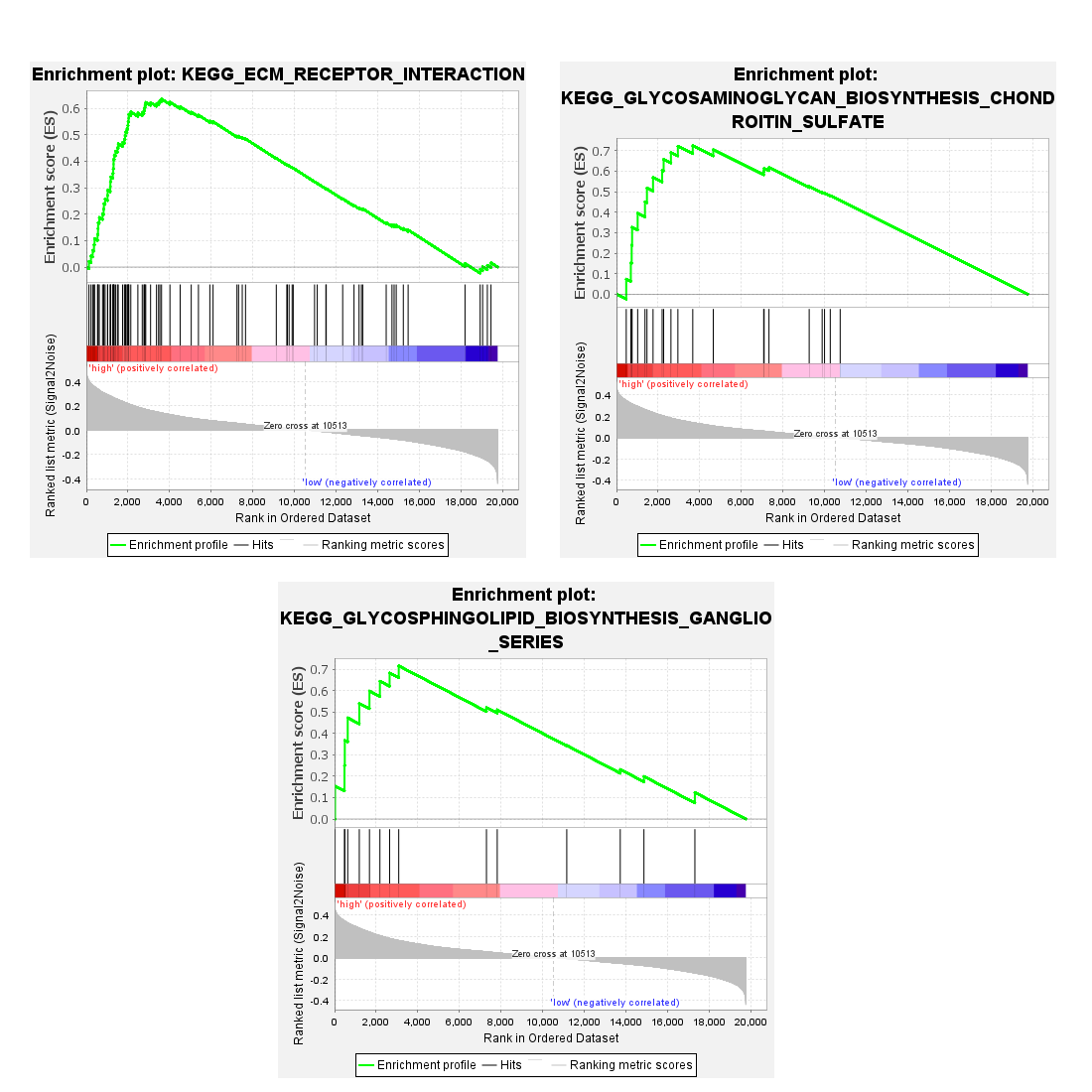

Supplement: Supplementary file 2 — Figure S2. [file JCMM-27-714-s004.png]
