# Supplementary material for: Integrative analysis of DNA methylation and gene expression through machine learning identifies stomach cancer diagnostic and prognostic biomarkers
Source: J Cell Mol Med. 2023 Feb 13;27(5):714–26. doi: 10.1111/jcmm.17693 (PMC9983314; doi:10.1111/jcmm.17693)
Supplement: Supplementary file 4 — Appendix S1. [file JCMM-27-714-s002.docx]

Figure S1. **Independence of four prognostic biomarkers in OS prediction.** Kaplan–Meier and ROC analysis were performed on patients stratified based on their stages **(A)**, gender **(B)** and age **(C)**. OS—overall survival; AUC—area under the curve; ROC—receiver-operating characteristic.

Figure S2. **GSEA analysis of low- and high-risk groups from TCGA-STAD cohort.** GSEA KEGG pathway enrichment for genes of high-risk samples. GSEA—gene set enrichment analysis; TCGA—The Cancer Genome Atlas; STAD—stomach adenocarcinoma; KEGG—Kyoto Encyclopedia of Genes and Genomes.
